# Extended prenatal and postnatal home visits in a vulnerable area in Sweden—a pilot study

**DOI:** 10.1080/02813432.2023.2277756

**Published:** 2023-11-29

**Authors:** Kuo Zhe Chin, Bertil Marklund, Sven Kylén, Sofia Dalemo

**Affiliations:** a Närhälsan Guldvingen Healthcare Centre, Lidköping, Sweden; b R&D Centre Skaraborg, Skövde, Sweden; c Public Health and Community Medicine, Institute of Medicine, Sahlgrenska Academy, University of Gothenburg, Gothenburg, Sweden; d R&D Centre Fyrbodal, Vänersborg, Sweden; e Centre on Health Care Improvement and Innovation, Chalmers University of Technology, Gothenburg, Sweden

**Keywords:** Child health services, health status disparities, house calls, maternal-child health centers, social support, Sweden, vulnerable populations

## Abstract

**Objective:**

Despite close to all-embracing access to child healthcare, health divides exist among children in Sweden. Home visits to families with new-born babies are a cost-effective way to identify and strengthen vulnerable families. An extended postnatal home visiting programme has been implemented in a disadvantaged suburb in Stockholm with positive results.

**Design:**

Longitudinal, prospective study and register study from medical records.

**Setting:**

A vulnerable rural area in Sweden.

**Intervention:**

A parent advisor from the social services and a midwife performed an extended home visiting programme during the end of pregnancy to mothers of children born between 1 May 2018 and 31 May 2019. During these children’s first 15 months, three additional home visits were made by a parent advisor and a child healthcare nurse. The aim of the study is to evaluate the effect of the intervention on the health of the children and the mothers.

**Subjects:**

All firstborn children at the study site (*N* = 30 study, *N* = 55 control group).

**Main outcome measures:**

The proportion participating in visits to the child and maternal healthcare services, children being breastfed and receiving childhood vaccinations.

**Results:**

There were fewer absentees in the study group during routine check-up visits (93 vs. 84%). More mothers in the study group attended the check-up with the midwives (90 vs. 80%). More children in the study group were breastfed (90 vs. 67%) and received all vaccinations (100 vs. 96%).

**Conclusion:**

Supplementing the extended home visiting programme with a visit at the end of pregnancy seems to contribute to fewer absentees at routine visits for both mothers and children; furthermore, more children were breastfed and vaccinated compared with the control group.

## KEY POINTS

Evaluation of four additional home visits to all firstborn children by parent advisors and healthcare professionals during the end-of-pregnancy period and early childhood in a vulnerable area showed the following benefits:

- More children in the study were breastfed and received all vaccinations compared with the control group.
- There were fewer absentees at the routine check-up visits to the child healthcare centre in the study compared with the control group.
- More mothers in the study group attended the check-up visits to the midwife 2 months after delivery compared with the controls.

## Introduction

In the past decades, overall health has improved in the population, although not for all groups. Differences in both physical and mental health exist between different socioeconomic groups [1]. Examples of social risk factors are parents with low education, growing up in poverty, and ethnic minority. Often, a family is affected by several risk factors simultaneously, which can be devastating. Children growing up in disadvantaged areas tend to suffer from adverse health, a higher risk of exposure to tobacco smoke, fewer infants are breastfed, and lower vaccination rates [2]. The Child Healthcare Service (CHS) offers free and close to all-embracing healthcare services for all children in Sweden [3]. However, there are still families that require additional assistance to ensure their children the same conditions as other children [4]. Both Finnish and American studies have shown positive effects up to young adulthood on the growth, development, and psychological status of the children after additional home visits to families during the child’s first years [5–7].

Based on the Swedish CHS programme, children are followed up by the CHS at least 16 times during their first 6 years of life [3]. Two of these follow-ups are home visits when the infant is 2 weeks and 8 months old. To achieve equity in society, Professor Marmot introduced the theory of proportional universalism. To reduce social disparities in health, actions must be universal, but with a scale and intensity proportional to the degree of disadvantage [8]. Based on this framework, the Swedish CHS operates on three tiers of childcare provision. In tier 1, a general healthcare programme is provided to all families. In tier 2, extended interventions are made by the given from CHS when needed. In the last tier, extra needs-based resources will be allocated to vulnerable families from another level of care, for example, the social services or psychologists [3].

All new mothers are offered a check-up by the midwife at the maternal healthcare centre about 2 months after delivery, when they can discuss their physical and mental health as well as contraception. Fewer foreign-born mothers attend the check-ups; 70% on a national level compared with 82% for Swedish-born mothers [9]. In Sweden, approximately 13% of women suffer from symptoms of depression in the first months after giving birth, which is slightly higher than during other periods in life [10]. Children’s development is negatively affected by parental depression [11], and postnatal maternal depression screening is offered at the CHS when the baby is 6–8 weeks old, according to the Edinburgh Postnatal Depression Scale (EPDS) [12].

Home visits from the CHS is a cost-effective way to identify [13] and support vulnerable families [7,14] at an early stage. They provide an insight into the environment families live in, which increases the understanding of families’ unique needs [4,7]. Parents feel more relaxed and secure during home visits [15], which contributes to strengthening their self-confidence, motivation, and parenting ability [7].

**Figure 1.**
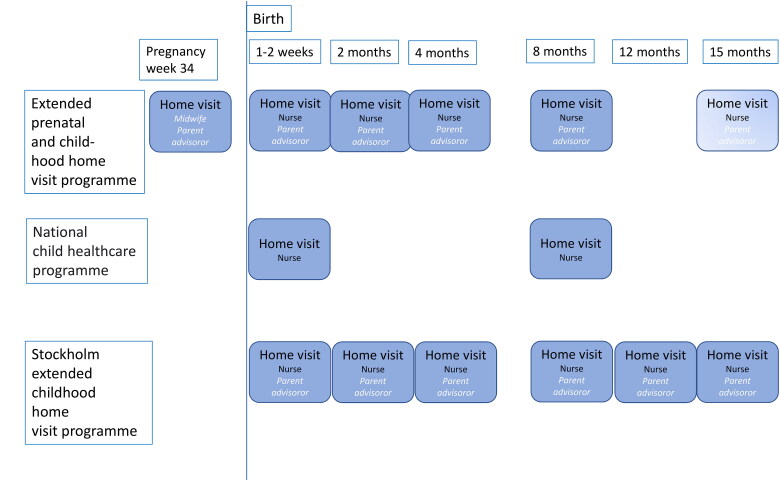
Flow chart showing the extended prenatal and childhood home visits made in this study, the national child healthcare programme in Sweden and the extended childhood home visiting programme in the Stockholm study [17].

In a disadvantaged suburb in Stockholm, an extended home visiting programme, including four extra home visits at 2, 4, 12, and 15 months of age, was offered to all first-time parents, se Figure 1 [16]. The home visits are carried out by a CHS nurse and a parent advisor, (trained social worker), from the preventive social services. The detailed content of the home visits is described elsewhere [15]. The children in the Stockholm study had a higher proportion of breastfeeding at 6 months (69 vs. 61%) and a higher vaccination rate (94 vs. 93%) than the control group [17,18]. The Swedish National Board of Health and Welfare therefore wanted to investigate whether a similar picture can be seen in rural areas or in less vulnerable communities [19], and recommended that the extended home visiting programme be supplemented by a home visit by a midwife and parent advisor at the end of the pregnancy [19]. The aim of the present study, carried out in a rural area, was to evaluate if there is an effect on the health of the child and the mother when the extended home visiting programme is supplemented by a home visit by a midwife and a parent advisor at the end of the pregnancy, measured as the proportion participating in visits to child and maternal healthcare, children being breastfed and receiving childhood vaccinations.

## Material and methods

The study was performed in a rural municipality in southwestern Sweden with 9000 inhabitants. The municipality was chosen for the intervention because it included families with lower socioeconomic status. There is only one healthcare centre in the municipality with a well-functioning family centre; a joint venue including an open preschool, a maternal healthcare centre, the CHS, and the social services.

### Intervention

Midwives at maternal healthcare centre invited parents who met the inclusion criteria to participate in the intervention during a prenatal assessment between the 29th and 32nd week of pregnancy. A midwife and a parent advisor from the social services carried out the first home visit during week 34 of the pregnancy, see Figure 1. Home visits were then offered in week 1–2 and when the child was 2, 4, 8 and 15 months old. The detailed content of the home visits is described elsewhere [15]. Many of the visits when the baby was 15 months would have taken place in the middle of the Covid-19 pandemic in 2020 when no home visits were carried out in the region. We have therefore chosen not to include this visit in the study, but only the first five home visits. Families who declined to participate in the intervention were offered standard care according to Swedish national CHS programme.

### Study participants

All families who lived in the municipality and were expecting their first child together in Sweden were included in the study. Both parents could have children born overseas and one previous child born in Sweden, but not both. The estimated birth date of the child should be between 1 May 2018 and 31 May 2019. During the study period, 38 mothers were enrolled in maternal healthcare. A total of four families declined to participate in the extended home visiting programme, two families miscarried, and two families moved from the municipality before the study was completed. As a comparison group, in this study called control group, children who went through the ordinary CHS programme were selected from the previous two years from the same healthcare centres. A total of five families from the control group were excluded as they did not attend the CHS programme from birth, which resulted in incomplete data. A total of 30 study and 55 control families participated.

### Data collection

Data were extracted from maternal healthcare records (Obstretrix, Oracle Cerner) until the check-up about 2 months after delivery and from CHS records (AsynjaVisph, CompuGroup Medical). The children’s records cover 18 months, including the visit for inoculation against measles, mumps, and rubella (MMR). The last visit in the study took place in August 2020. Variables that were noted were need of a language interpreter and the parents’ country of birth. If the parents came from different countries, the one who did not come from Sweden was noted. No family had parents born outside Sweden in two different parts of the world. Participation in the CHS programme and breastfeeding and MMR vaccination of the children were also noted. Finally, the mothers’ check-up by the maternal healthcare service and contraceptive use were registered. The EPDS is administered at the child healthcare centre 2 months postpartum [3] and translated into 22 languages. A total of 12 points in the EPDS is the cut-off level [3].

### Region Västra Götaland

Another data collection exercise was carried out, from the whole region where the rural municipality is situated in Region Västra Götaland. The region is Sweden’s second largest with a population of 1.8 million people and includes both a large city, Gothenburg, and rural areas. The region is sometimes described as Sweden in miniature. Figures from the annual report of the central child healthcare service in Region Västra Götaland and from the Swedish Pregnancy Register were included in the tables when it was possible to obtain them.

### Data analysis

The collected data were primarily compared between the study and control group. The study is a total survey of the entire target population. Therefore, no analyses to obtain p values have been carried out.

As a further broad general comparison, figures from the entire region of Västra Götaland, both privately and publicly run CHS centres, and the entire target population in the Stockholm study, were compared [17].

## Results

Table 1 shows that the mean age of the mothers in the study group was higher than the controls, who included four teenage mothers. Most families in both groups had their first child during the studied periods. The figures for at least one smoking parent in the study group and the control group were 63% and 23%, respectively. It was mainly the fathers who smoked. Many parents were born in countries other than Sweden, especially in the study group. The frequencies of parents needing a language interpreter was around 20% in both groups, Table 1.

**Table 1.** Background characteristics of study and control families in a vulnerable area in Sweden.

	Study		Control		Region*		Stockholm	
Year	2018–2019		2016–2018		2018		2013	
	Medical records				Register		Article [17]	
Data source	Number	(%)	Number	(%)	Number	(%)	Number	(%)
*Total*	30		55		19 268		99	
*Age, mean, range (year)*								
Mothers	34 (20–45)		25 (16–39)		31		26	
Fathers	38 (20–55)		40 (20–59)		34		–	
*Parental origin*								
Sweden	15	(50)	40	(72)	–	(63)		(8)
Europe	0	–	2	(4)	–	(12)		(6)
Middle East	11	(36)	8	(14)	–	(9)		(15)
Africa	2	(7)	1	(2)	–	(7)		(55)
Asia	2	(7)	3	(5)	–	(6)		(11)
Other					–	(3)		
Unknown	0		1	(2)				(4)
*At least one smoking parent*	19	(63)	13	(23)	–	(10)		
*Need of language interpreter*	7	(23)	11	(20)		(3)		
*Earlier children in the family*								
0	23	(77)	48	(87)				
1	3	(10)	4	(7)				
>1	4	(13)	3	(5)				

*Note.* *Region Västra Götaland 1.6 million inhabitants.

**Figure 2.**
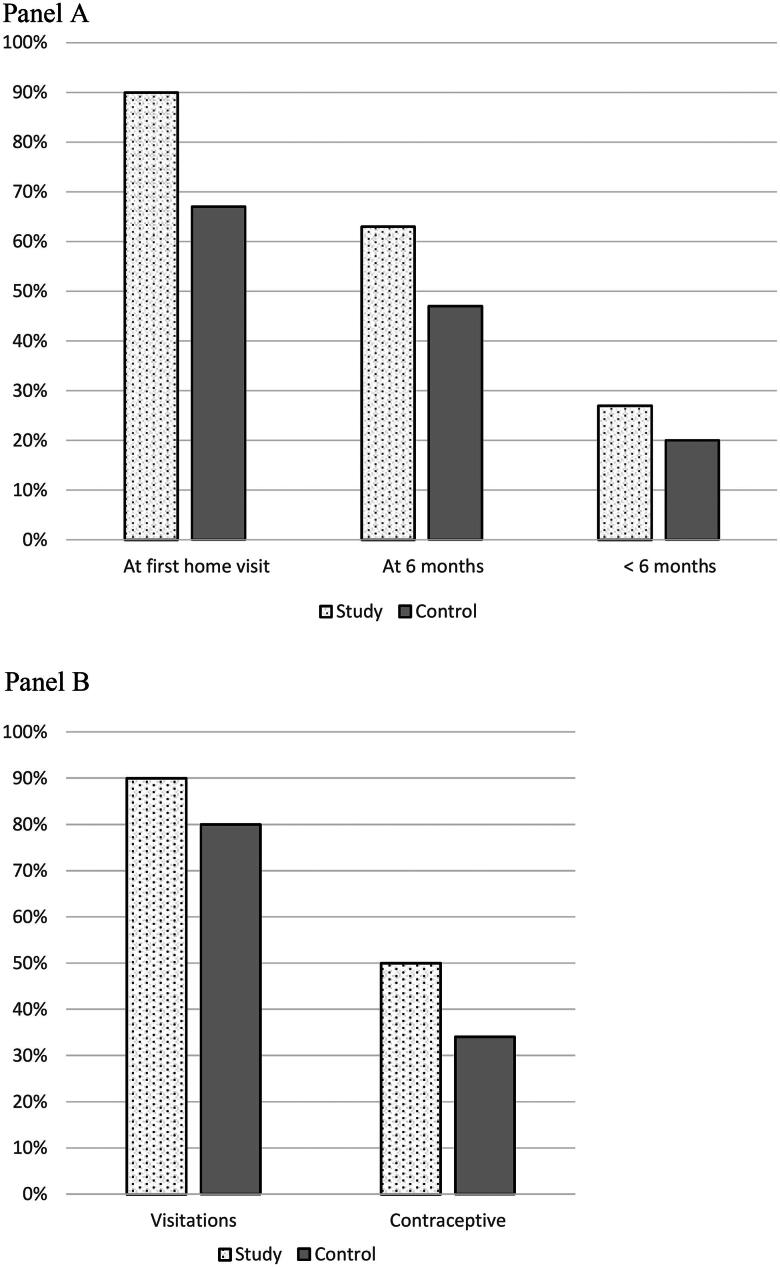
Panel A the proportion of children breastfeeding in the study and control groups at the first home visit, at age 6 months and after 6 months. Panel B The proportion of mothers in the study and control group participating in follow-up check-up to maternal healthcare about two months after delivery and that used contraceptives not including condoms.

All children in the study group received all vaccinations according to the Swedish vaccination programme. Four per cent of the children in the control group were unvaccinated, Table 2. The frequency of breastfeeding was higher in the study group compared with the controls, 90% vs. 67% at the first home visit and 63% vs. 47% at 6 months, Figure 2. Almost all families in the study group received all five home visits or missed only one. Regular appointments for examinations and controls are part of the CHS programme. ‘No missed appointments’ were fewer in the study group with an attendance rate of 93% compared with 84% for the controls, Table 2.

**Table 2.** Postnatal characteristics of study and control families in a vulnerable area in Sweden.

	Study		Control		Region*		Stockholm	
Year	2018–2019		2016–2018		2018		2013	
		Medical records			Register		Article [17]	
Data source	Number	(%)	Number	(%)	Number	(%)	Number	(%)
*Total*	30		55		19 268		99	
*Vaccination*								
Basic vaccinations^†^	30	(100)	53	(96)	–	(97)		(94)
MMR‡	30	(100)	53	(96)	–	(97)		(80)
Unvaccinated	0		2	(4)	–	(2)		
*Breastfeeding*								
at first home visit	27	(90)	37	(67)	–		–	
at 6 months	19	(63)	26	(47)	–	(62)	–	(69)
after 6 months	8	(27)	11	(20)	–	(28)	–	(49)
*Completed home visits*								
1	0		2	(5)	–	(38)	0	
2	0		51	(92)	–	(41)	6	
3	2	(7)	–		–		7	
4	10	(33)	–		–		8	
5	18	(60)	–		–		30	
6					–		47	
Missed visitations at the CHC*								
0	28	(93)	46	(84)	–		–	
1	1	(3)	5	(9)	–		–	
2	0		1	(2)	–		–	
3	1	(3)	3	(5)	–		–	

For comparison, figures from the whole of Region Västra Götaland and the Stockholm study are also reported.

*Note.* ^†^Basic vaccinations: diphtheria, whooping cough, tetanus, polio, Hib, hepatitis B (3, 5, 12 months), pneumococcus (3, 5, 12 months).

^‡^Measles, mumps, rubella (18 months), *Child Healthcare Service.

More mothers in the study group underwent the routine postnatal depression screening using the EPDS at the CHS; 90% compared with 65% in the control group, Table 3. There was no difference in the percentage that exceeded the cut-off level in the groups. More mothers in the study group than in the control group came for the return visit to the maternal healthcare care centre; 90% vs. 80%, Figure 2. Contraceptive use was also more frequent in the study group compared with the control group, 50% vs. 34%, Table 3, Figure 2.

**Table 3.** Postnatal characteristics of mothers in study and control families in a vulnerable area in Sweden.

	Study		Control		Region	
Year	2018–2019		2016–2018		2018	
	Medical		Records		Register	
Data source	Number	(%)	Number	(%)	Number	(%)
*Total*	30		55		19,268	
*Child healthcare centre*						
EPDS^†^						
Completed	27	(90)	36	(65)	–	(80)
Above cut-off level	2	(7)	4	(7)	–	
*Maternity care centre*						
Postnatal visits to midwife						
Attended	27	(90)	44	(80)	–	(80)
Unknown	–		5	(9)	–	
Contraceptive^‡^						
Use	15	(50)	19	(34)	–	
Unknown	–		5	(9)	–	

*Note.* ^†^Edinburgh Postnatal Depression Scale.

^‡^Not including condom.

Figures from the whole of Region Västra Götaland are also reported for comparison.

## Discussion

### Statement of principal findings

A unique point of interest in this pilot study is that the programme includes a visit by a midwife and a parent advisor at the end of the pregnancy. Otherwise, it is based on the programme in Stockholm, which has shown good results in studies [18]. A remarkably large percentage of the children in the study group, 90%, were breastfed at the first home visit and all the children received all vaccinations. This pilot study, therefore, speaks för that a home visit before the birth of the child, when breastfeeding can be discussed in a relaxed environment, seems to be valuable [14,15]. The programme further seems to contribute to fewer absentees at routine visits for both mothers and children. This result should be followed up in new larger studies.

### Strengths and weaknesses of the study

A strength of the study is that all families who had their first child in Sweden in the area were included. No family needed to feel singled out, as everyone was invited. The results can therefore more easily be generalised without any statistical uncertainty about the sample.

The studied area can be characterized as socially vulnerable according to common parameters in this context such as the percentage of unemployed people, the level of education, the burden of care measured as the Care Need Index. Common parameters to compare in this CHS context are children who have at least one parent who smokes and teenage mothers [6,20]. For the entire studied group, 38% of the children had at least one smoking parent, and teenage mothers made up 5% of the sample. The corresponding figures for Sweden are 11 and 0.4%, respectively [3,21].

A child is affected by the environment it lives and grows up in. It is not possible to exclude a child from its environment to study children’s development and health. Each area is unique regarding its type of socioeconomic burden, and we believe it is a great advantage that the comparison group, (control group) came from the same area and from the period before the study was conducted. Furthermore, staffing at the CHS was stable and the same nurses worked both during the study and in the control group.

A further strength is that improved values were seen in the study group despite poorer background values on socioeconomic parameters, such as parents born overseas and smoking, compared with the control group.

This pilot study is small. However, the participation rate in the study group is high at 79%.

Another weakness of the study is that we are comparing a study group with older mothers and a control group with younger mothers. This may of course have had an impact on the results. Older mothers may have appreciated the home visits more and been more receptive. However, most previous studies have been performed on younger mothers [6]. The age of the mothers in the study group is, however, on par with the average age of mothers in the region, and the control group is comparable to the average age of the participants in the Stockholm study [17]. Therefore, none of the average ages appear to be outliers, but perhaps more an expression of the study being small.

Fewer parents in the study group during 2018/2019 were born in Sweden compared with the control group during 2016/2018. The figures can be partially explained by the fact that the municipality has received many refugees. In 2015, it was one of the municipalities in Sweden that received the largest number of refugees per thousand inhabitants [22]. It can be seen as a strength of the study that despite there being more parents from other countries with language difficulties, for instance, in the study group, the intervention seemed to have an effect.

Due to the Covid-19 pandemic, some home visits were not carried out as planned, as circumstances would not allow it. This may have caused some disparity in the effectiveness of the programme.

### Findings in relation to other studies

#### Low socioeconomic status

Previous review study has pointed out that home visits can produce replicable effects on children’s health and development when targeting on populations that need, want, and can benefit from the service [14]. Families in many other studies also live in an area with low socioeconomic status [6,18]. The frequency of parents needing translator services, 20% in both groups, also indicates that the families involved were not yet fully integrated in Swedish society [23]. The high percentage of smokers can relate to the high percentage from other countries because Sweden from an international perspective successive has taken wide-ranging action to reduce smoking [1]. Both this study and the Stockholm study were performed in vulnerable areas with low socioeconomic status, and both achieved higher MMR vaccination coverage as a result of the increased number of home visits [18]. However, a review study of women with substance abuse problems, however, found no difference between the groups that received home visits and the comparison group [24].

#### Collaboration between professionals

An important success factor seems to be the collaboration between midwives, parent advisors, and child healthcare nurses [25]. The well-functioning family centre is an important meeting point that already before the start of the project included a maternal healthcare centre, the CHS, the social services, and an open preschool in one physical location [26]. The Swedish police also highlights the fact that early preventative cooperation between various social functions, such as the CHS and the social services, contributes to achieving long-term goals such as reducing crime in vulnerable areas [27].

Increased home visits lead to a higher participation rate by the families at follow-up visits to both midwifes and the CHS. For the professionals, home visits are more time-consuming due to the time of transport than regular visits to the CHS. It appears that some of this time can be compensated for by fewer families missing their regular CHS visits.

#### Pilot study

The study is a pilot, but an important one as it was carried out just before the covid-19 pandemic. Due to the pandemic, many other similar larger studies have been postponed. Unfortunately, it was not possible to carry out all the home visits when the children were 15 months old, as some were cancelled due to the pandemic. However, the last (sixth) home visit is perhaps the least important. Many children in Sweden have started preschool at 15 months, and the family is then at a different stage in life when they may not be as impressionable and receptive to advice at a home visit. The earlier home visits seem to be more important for creating relationships between the families and the various professionals [15].

#### Children

As many as 90% of the children in the study group were breastfed at the first home visit. However, it seems that more mothers in the study group both start and continue to breastfeed [17]. The percentage in the study group of mothers breastfeeding when the baby is 6 months old is at the same level as in both the Västra Götaland region and the Stockholm study [18], while the control group is at a lower level.

Extended home visits supplemented with a visit during the end of the pregnancy covaries with both a higher rate and the duration of breastfeeding. It seems to be valuable to make a home visit before the birth when breastfeeding can be discussed in a relaxed environment. In many cases, it is too late to discuss breastfeeding in connection with the home visit when the baby is 1-2 weeks old [28]. This result should be followed up in new larger studies.

No children in the study group were unvaccinated, which is otherwise a phenomenon that occurs in a certain percentage of the children at the CHS in Sweden, Table 2 [29]. This may be an expression of success regarding one of the aims of the intervention, namely, to promote trust in antenatal care and the CHS as well as the social services.

#### Mothers

It is valuable if interventions aimed at socioeconomically vulnerable families have an effect also when the children grow up. Assessment of the mothers’ mental well-being using EPDS screening is crucial [11]. More mothers undergoing EPDS screening may contribute to finding more mothers with depression. Depressed mothers affect their children’s development and their risk of developing depression [30].

For many families, the mothers attending the follow-up check-up at the maternal healthcare centre may be of great value. Mothers that wish can then have contraceptives prescribed and those who have had complications in connection with childbirth can get help with these.

### Meaning of the study

The home visit at the end of the pregnancy seems to have contributed to many excellent results being achieved with regard to breastfeeding, vaccinations and families participating in visits to child and maternal healthcare centres in a socioeconomically vulnerable area. An important contributing factor was the successful cooperation between healthcare professionals and parent advisors. This early preventative effort is relatively cheap compared with curbing the development of crime in society [5–7] but is of considerable value for families that require additional assistance to ensure the same conditions for their children as for other children. The transition to this way of working would not be impossible for other regions in Sweden as home visits are already included in the ordinary CHS programme. However, the logistics when parent advisors are also involved may be a challenge. We look forward to following these children to evaluate whether the intervention may also have a long-term effect on school results, as was seen in the Finnish and American studies [5–7].
