# Correction

**DOI:** 10.1080/02813432.2023.2286059

**Published:** 2023-11-29

**Article title:** “Extended prenatal and postnatal home visits in a vulnerable area in Sweden—a pilot study”

**Author:** K. Z. Chin, B. Marklund, S. Kylén and S. Dalemo

**Journal:** *Scandinavian Journal of Primary Health Care*

**DOI:** 10.1080/02813432.2023.2277756

When this article was first published online, incorrect text “Completed home visits” in first column of Table 2 was used. This has now been corrected with the correct text “Missed visitations at the CHC*” as shown below:

**Table 2.** Postnatal characteristics of study and control families in a vulnerable area in Sweden.

	*Study*		*Control*		***Region*** ^*^		*Stockholm*	
*Year*	*2018–2019*		*2016–2018*		*2018*		*2013*	
*Data source*		*Medical records*			*Register*		*Article [17]*	
	Number	(%)	Number	(%)	Number	(%)	Number	(%)
*Total*	30		55		19 268		99	
*Vaccination*								
Basic vaccinations^†^	30	(100)	53	(96)	–	(97)		(94)
MMR^‡^	30	(100)	53	(96)	–	(97)		(80)
Unvaccinated	0		2	(4)	–	(2)		
*Breastfeeding*								
At first home visit	27	(90)	37	(67)	–		–	
At 6 months	19	(63)	26	(47)	–	(62)	–	(69)
After 6 months	8	(27)	11	(20)	–	(28)	–	(49)
*Completed home visits*								
1	0		2	(5)	–	(38)	0	
2	0		51	(92)	–	(41)	6	
3	2	(7)	–		–		7	
4	10	(33)	–		–		8	
5	18	(60)	–		–		30	
6					–		47	
*Missed visitations at the CHC* *								
0	28	(93)	46	(84)	–		–	
1	1	(3)	5	(9)	–		–	
2	0		1	(2)	–		–	
3	1	(3)	3	(5)	–		–	

For comparison, figures from the whole of Region Västra Götaland and the Stockholm study are also reported.

*Note*. ^†^Basic vaccinations: diphtheria, whooping cough, tetanus, polio, Hib, hepatitis B (3, 5, 12 months), pneumococcus (3, 5, 12 months).

^‡^ Measles, mumps, rubella (18 months).

* Child Healthcare Service.

